# MicroRNA-107 is a novel tumor suppressor targeting POU3F2 in melanoma

**DOI:** 10.1186/s40659-020-00278-3

**Published:** 2020-03-14

**Authors:** Guizhi Zhao, Zhili Wei, Yang Guo

**Affiliations:** 1 Department of Dermatology, Daqing Oilfield General Hospital, No. 9 Zhongkang Road, Saertu District, Daqing, 163000 Heilongjiang China; 2 Department of Stomatology, Daqing Oilfield General Hospital, No. 9 Zhongkang Road, Saertu District, Daqing, 163000 Heilongjiang China; 3 Department of Dermatology, Dongzhimen Hospital, Beijing University of Traditional Chinese Medicine, No. 5 Shipping Warehouse, Dongcheng District, Beijing, 100700 China

**Keywords:** Melanoma, miR-107, POU3F2, SH-4, Metastasis

## Abstract

**Background:**

Melanoma is one of the major types of skin cancer. The metastatic melanoma is among the most lethal forms of malignant skin tumors. We hereby aimed to characterize a novel microRNA (miR) in the metastatic melanoma model.

**Methods:**

First, we evaluated the expression of miR-107 in melanoma cells and tumor tissues. The comparison between primary and metastatic cancer tissues was also accessed. Next, we examined the impact of miR-107 on melanoma cell proliferation, cell cycle, colony formation, apoptotic activity, migration and matrix invasion. A downstream target of miR-107 was also predicted and validated functionally in melanoma cells.

**Results:**

Our findings showed miR-107 was significantly downregulated in melanoma. Its expression was lowest in metastatic form. Over-expression of miR-107 reduced melanoma cell proliferation, migration and invasion. POU3F2 was identified as the downstream target of miR-107. Over-expression of POU3F2 antagonized miR-107-mediated inhibitory effect on melanoma cells.

**Conclusion:**

Our study has reported miR-107 as a novel tumor suppressive factor in the metastatic melanoma model. It has provided new avenue to manage melanoma and improve the survival rate in the advanced stage.

## Background

Melanoma is one of the major forms of skin cancer, representing about 5% of all cutaneous malignancies. The worldwide incident rate of cutaneous melanoma is about 3 per 100,000 persons [1]. The tumor was initially derived from melanocytes, a special type of skin cells found in the basal layer of epidermis that produces melanin. Melanoma can be grouped into different stages depending on their occurring place and spreading status. In earlier stages (0 and 1), the melanoma is still largely found in the epidermis. This forms the primary tumor site. At stage 2, the tumor begins to invade into the dermis and may have potential to migrate. At the advanced stages, melanoma becomes very metastatic. It can infiltrate into the lymphatic and blood systems and eventually spread out to other tissues and organs such as the lung, bone, liver and central nervous system. The metastatic melanoma is very difficult to manage and the estimated survival rate for 5-year is less than 20%, placing it as one of the most lethal forms of skin cancer [2, 3].

The biggest risk factor for melanoma incidence is sun exposure. Population with fairer skin or living in places with higher UV ray exposure may have increased risk of developing melanoma. Murine models have been used to demonstrate the causative link between UV-induced melanogenesis and melanoma formation. Frequent DNA damages in the skin caused by prolonged UV exposure may also be a mechanism that triggers the melanocytes’ cancerous transformation. In addition to sun exposure, other environmental factors such as smoking, chemicals exposure in the workplace such as heavy metals, or certain drug usages have been associated with melanoma in recent epidemiological studies [4, 5]. Genetic factors can also increase the risk of developing melanoma. For instance, somatic mutations in CDKN2A and BRAF are commonly found in the sporadic form of melanoma. In the inherited form of melanoma, the primary genes involved are CDKN2A and MC1R. MC1R is one of the key regulators in the melanogenesis process of melanocytes, implicating a strong role of melanogenesis pathway in melanoma tumorigenesis. However, in most cases, the development of melanoma is contributed by the combinational changes and interplays of environmental and biological factors [6, 7].

Epigenetic regulation such as dysfunctional microRNA (miRNA, miR) expression is found to be implicated in the process of malignant melanoma [8–10]. MiRNAs are a class of short non-coding RNAs with about 22 nucleotides. They regulate the gene expression at post-transcriptional level by targeting the 3′UTR of the target gene transcripts. One miRNA may target several gene transcripts and a 3′UTR may contain several different miRNA binding sites. Therefore, a network of miRNAs acts in an orchestrated manner to fine tune the cell transcriptome. They are found to be involved in multiple cellular processes including division, growth, differentiation, migration and apoptosis. The abnormal miRNA expression is also implicated in the pathogenesis of multiple cancers such as in the lung, liver, pancreas, brain and skin, contributing to tumor initiation, development and metastasis [11, 12]. Initial study on miRNA expression in melanoma was reported in a comprehensive profiling investigation on 217 mammalian miRNA expressional pattern from 334 tumor samples including melanoma, which gave an insightful evidence on the potential roles of miRNAs in melanoma [13]. The first study to propose a role of a single miRNA in the tumorigenesis of melanoma was published in 2008, where the authors identified that miR-137 targeted Micropthalmia-associated transcription factor (MITF), a key regulator of melanocyte proliferation, maturation and pigmenting process, to regulate the malignant transformation of melanocytes [14].

In our study we sought to characterize a novel miRNA in melanoma. We first associated its expression with melanoma cell lines and clinical tissues. Functional studies in cell proliferation, apoptosis and migration were carried out in melanoma cells derived from metastatic tumors. We also validated one downstream target of the miRNA in melanoma cells suggesting a mechanism contributing to the melanoma formation and migration.

## Methods

### Cell culture

Human melanoma lines SK-MEL-1, A375, G-361, SK0MEL-3, SH-4 and SK-MEL-24 were purchased from ATCC. They were grown in RPMI medium supplemented with 10% fetal bovine serum (FBS) and 50 mM l-glutamine at 5% CO_2_/37 °C. Primary melanocyte cells were ordered from Lonza (adult donors). They were grown in Medium 254 (Thermo Fisher Scientific) supplemented with human melanocyte growth supplement (#S0025, Life Technologies) at 5% CO_2_/37 °C.

### Clinical specimens

Clinical measurement of miR-107 expression in human nevi and melanoma tissues was approved by hospital ethical committee. The specimens were harvested from the patients during the surgical removal with inform consent obtained. The expression level of miR-107 was determined by real time-PCR. The protein expression of POU3F2 was determined by ELISA kit from Mybiosource (Cat. MBS2881624) according to the manufacturer’s instruction.

### Real time-PCR analysis

Total RNA was isolated using Trizol method. 500 mg of total RNA was reverse transcribed with high capacity cDNA archive kit (Applied Biosystems). Quantification of miR-107 and U6 was done by SYBR green real-time PCR. The target primers were purchased from Qiagen. The real-time PCR was carried out on Biorad real-time PCR platform.

### miRNA mimic and transfection

miRNA mimic and negative control were ordered from MISSION^®^ microRNA Mimic platform. They were functionally tested with minimal off-target effects. Cell transfection of miRNAs was achieved with Lipofectamine 2000 (Invitrogen) according to the standard protocol.

### Cell proliferation assay

The cell proliferation rate was assessed with Cell Counting Kit-8 (CCK-8) according to manufacturer’s protocol. Briefly, cells were seeded 96-well plates at a density of 5000 cells per well. CCK-8 reagent was added 2 h before the measurement at 24, 48, 72 and 96 h post transfection. The cell proliferation curves were plotted by measuring the absorbance at 450 nm in a plate reader.

### Cell migration assay

Melanoma cells (5 × 10^4^ cells/well) were seeded and the wound closure measurement was done with Oris Cell Migration Assay (CMA5.101) following the manufacturer’s manual. Briefly, the cells were allowed to grow into confluency and Oris Cell Seeding Stopper was removed to create a wound. The open wound area was captured with a microscope under bright field and measured by ImageJ every 24 h for 3 days. The percentages of the open wound were plotted against the time points to calculate the wound closure rates.

### Cell invasion assay

The cell invasive potential was performed in CytoSelect™ 24-Well Cell Invasion kit (CellBiolabs) according to the manufacturer’s standard protocol. Briefly, cells were seeded at 3 × 10^4^ cells/well in the upper chamber onto a type I collagen-based matrix. Cell culture medium supplemented 10% FBS was used as a chemoattractant in the lower chamber.

### Colony formation assay

Melanoma cells transfected with miRNAs were seeded at 300 cells/well in a 6-well plate. They were allowed to grow for a week and then stained with 0.1% crystal violet. Total number of colonies (with more than 50 cells) was counted microscopically.

### Cell cycle analysis

The cells were seeded in 6-well plate with 4 × 10^5^ cells/well 24 h before the miRNA transfection. After 48 h, the cells were harvested by trypsinization, washed with phosphate-buffered saline and fixed in 70% ethanol. The cell cycle analysis was carried out using Cycletest™ PLUS DNA Reagent Kit (BD Biosciences) following the manufacturer’s manual. The data was acquired in NovoCyte flow cytometer.

### Luciferase assay

The 3′UTR sequence of POU3F2 containing miR-107 targeting site (Fig. 4a) were cloned downstream of the firefly luciferase gene in the vector (pMIR-REPORT) as named WT. Mutated 3′UTR sequence of POU3F2 complementary to miR-107 was cloned in the same vector as named MUT (Fig. 4a). Cells were transfected with WT or MUT reporter plasmids with either miR-107 or scramble control. The Renilla Luciferase vector (pRL-SV40) was co-transfected for normalization. The luciferase activities were quantified using Dual-Luciferase^®^ Reporter (DLR™) kit following the manufacturer’s instruction.

### Western blot analysis

Cells were harvested in RIPA lysis buffer supplemented with proteases inhibitors cocktail (Roche). Protein concentration was measured by Bradford assay. 30 µg protein sample was denatured in LDS sample buffer (Life Technologies) with heating at 75 °C for 5 min. The denatured samples were then fractionated in 12% SDS/PAGE, transferred to a nitrocellulose membrane, and blocked in 5% non-fat milk for 1 h at room temperature. Membranes were next incubated with the primary antibodies in 1:1000 dilution, followed by incubation with a secondary antibody (horseradish peroxidase-conjugated). The target proteins were detected with SuperSignal West Pico PLUS chemiluminescent kit on a Chemidoc (Bio-Rad) imaging system. PARP1 (ab194586), Caspase 3 (ab13847) and GAPDH (ab9485) antibodies were obtained from Abcam. Cytochrome c (A-8) antibody was from Santa Cruz Biotechnology. POU3F2 (D2C1L), CyclinD1 (92G2) and p21 (12D1) antibodies was from Cell Signaling. The protein signal was quantified by densitometry using ImageJ software.

### Statistical analysis

Statistical significance of a two-group comparison was analysed by unpaired Student’s t test. If more than two groups were compared, the statistical differences were calculated by one-way ANOVA analysis. Only p < 0.05 was regarded as statistically significant.

## Results

**Fig. 1:**
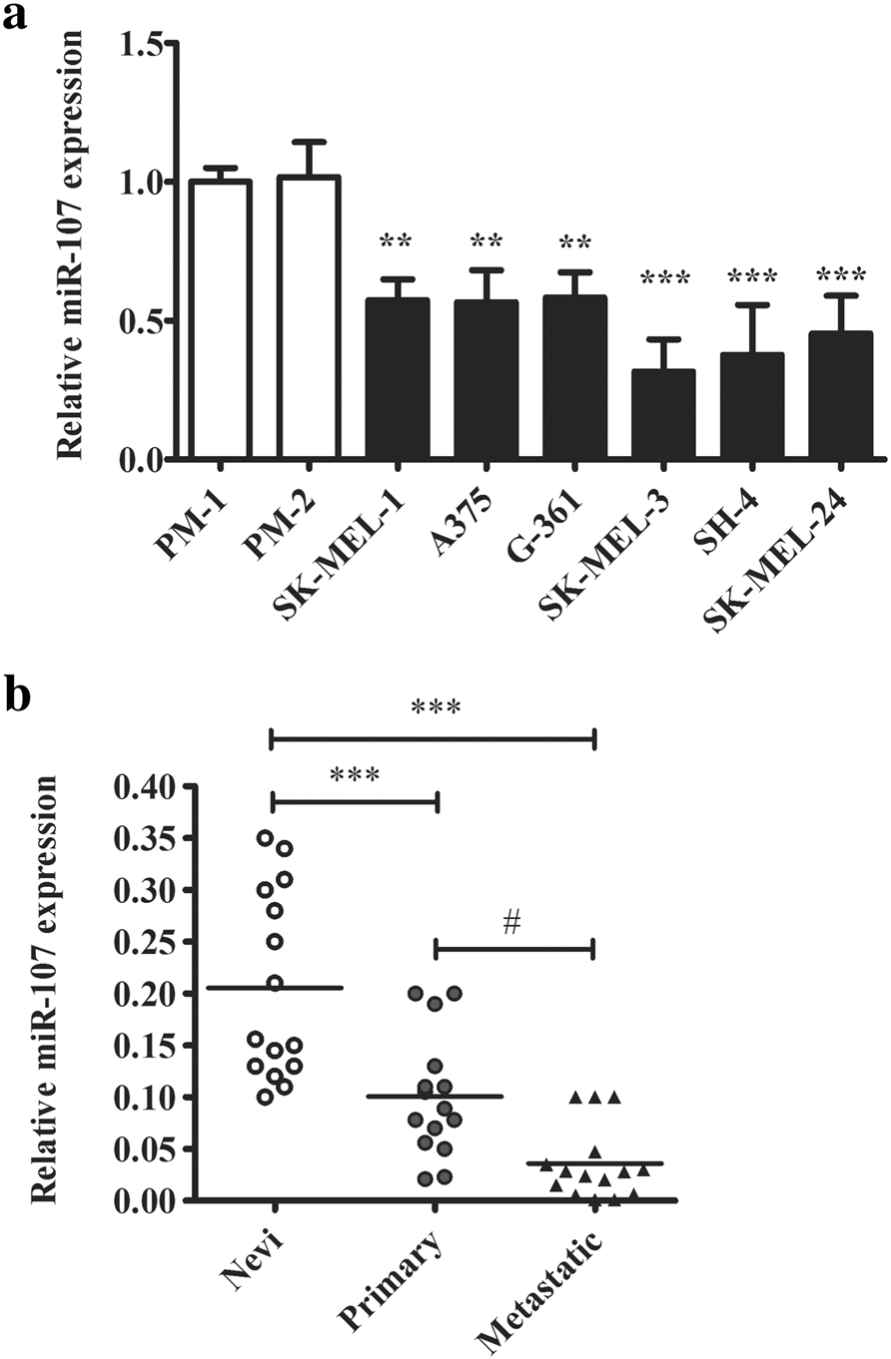
miR-107 is under-expressed in human melanoma cell lines and tumors. **a** The comparison of miRNA-107 expression in melanoma cancer lines (SK-MEL-1, A375, G-361, SK-MEL-3, SH-4, and SK-MEL-24) and primary melanocytes (PM-1, PM-2) from healthy donors. **b** The comparison of miRNA-107 expression in nevi, primary and metastatic melanoma human samples. The miRNA was quantified by realtime PCR. The data were presented as mean ± SD of at least three independent experiments (**p < 0.01, ***p < 0.001, as compared with control cells or nevi tissues; ^#^p < 0.05, as compared with primary tumors)

To study whether miR-107 was functionally implicated in melanoma, we first measured its expressional level in different melanoma lines including (SK-MEL-1, A375, G-361, SK-MEL-3, SH-4, and SK-MEL-24). Primary melanocytes (PM-1, PM-2) harvested from two healthy donors were used as base line control. As shown in Fig. 1a, the expression of miR-104 was consistently downregulated in melanoma cells. Interestingly, SK-MEL-3, SH-4 and SK-MEL-24 were derived from metastatic melanoma tissues. There was a further downward tendency of miR-107 expressional level in these lines as compared with A375, G361 and SK-MEL-1 harvested from non-metastatic sites. To confirm this hypothesis, we moved on to evaluate miR-107 expressional level in tumor tissues. We compared miRNA expression among three clinical sample groups: nevi from non-tumor tissues, primary melanoma and metastatic melanoma. As shown in Fig. 1b, miR-107 was significantly repressed in both primary melanoma and metastatic tumor. And the mean expressional level of miRNA-107 was lowest in metastatic group. Both cell and tissue studies suggested miR-107 might carry a novel function in melanoma and be implicated in the transition from tumor formation to metastasis stage of melanoma.

**Fig. 2:**
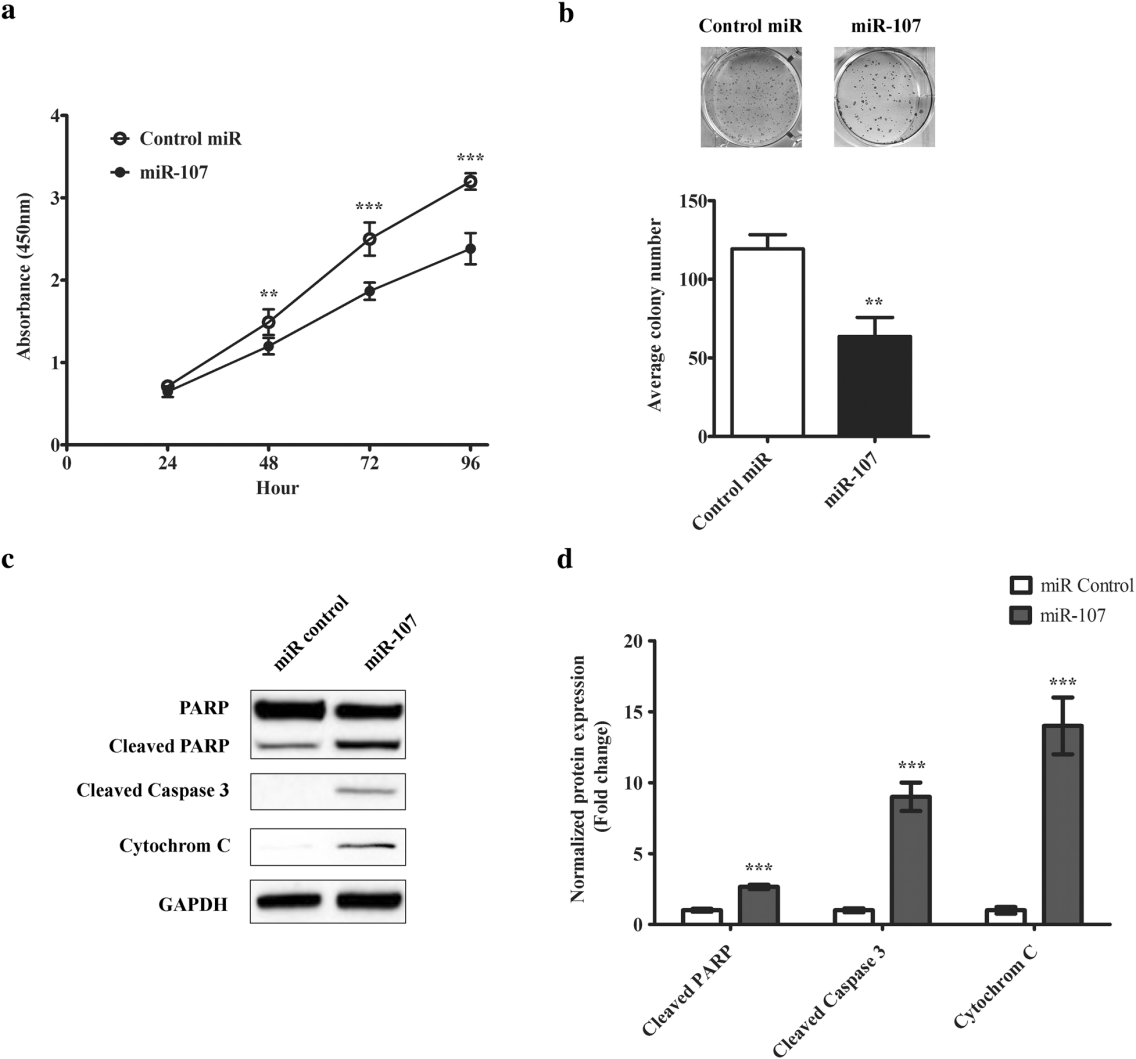
miR-107 inhibits melanoma cell proliferation, colony formation and induces apoptosis. **a** Cell proliferation study of SH-4 cells transfected with either miR-107 or the scramble control. The growth rate of cells was monitored using CCK-8 kit for 72 h. **b** Colony formation study of SH-4 cells transfected with either miR-107 or the scramble control. The colony formation ability of the transfected cells was accessed by counting the number of colonies (more than 50 cells) in the culturing plates. **c** Western blotting analysis of apoptotic pathway activities in transfected cells. The representative image from at least three independent experiments was shown. **d** The densitometric analysis of cleaved PARP1, cleaved Caspase 3 and Cytochrome C expression was shown. The data were presented as mean ± SD of at least three independent experiments (**p < 0.01, ***p < 0.001 as compared with control)

Next, we moved on to characterize the role of miR-107 in melanoma cells. Since miR-107 expression was strongly associated with late stage of melanoma, we chose SH-4, a metastatic melanoma line as our model cell line. First, we expressed miR-107 mimic in SH-4 and checked the impact on cell proliferation. As shown in Fig. 2a, the over-expression of miR-107 largely decreased the proliferative potential of cancer cells. The difference was first observed in 48 h after transfection. Then colony formation assay was used to further confirm this finding. As shown in Fig. 2b, there were much less colonies formed from cells transfected with miR-107, confirming our observation in cell proliferation assay. We also examined the impact of miR-107 on cell cycle regulation. As shown in Additional file 1: Figure S1A, B, Cyclin D1, a key regulator for G1-S transition, was downregulated in miR-107 transfected cells. In comparison, p21, a cell cycle inhibitor, was upregulated. Consistent with this observation, we also found that miR-107 expressing cells exhibited a G1 arrest pattern by flow cytometric analysis (Additional file 1: Figure S1C). In line with the suppression on cell proliferation, apoptotic pathway was found activated by miR-107 transfection. As shown in Fig. 2c, d, the expression of miR107 increased the cleaved forms of PARP and Caspase-3 and cytoplasmic cytochrome C expression, suggesting the canonical apoptosis program was activated.

**Fig. 3:**
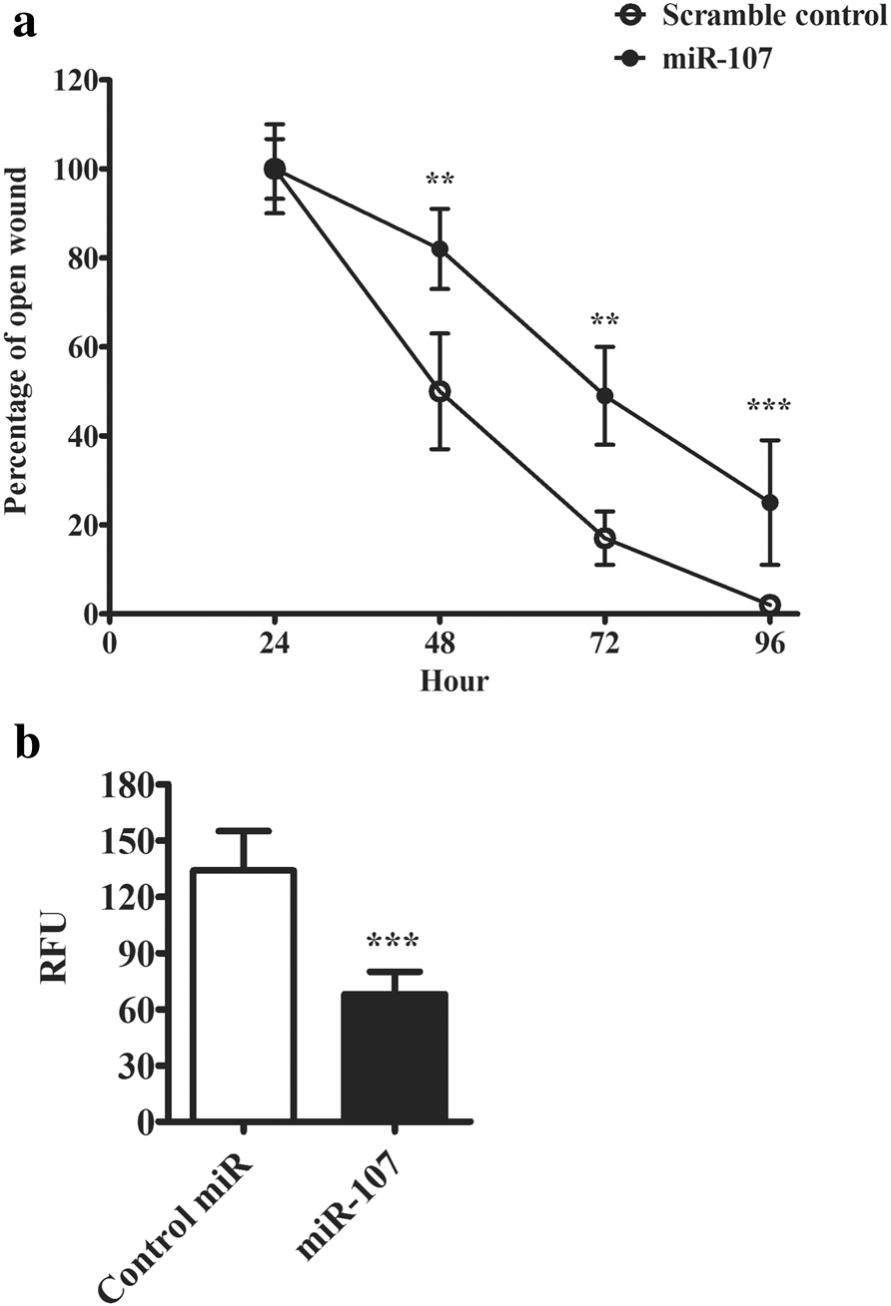
miR-107 inhibits migratory capacity of melanoma cells. **a** Graphic summary of cell migration rate in wound closure experiment. An open wound was created in the centre of a confluent monolayer of transfected cells. The percentage of the remaining open wound was recorded every 24 h for 4 days. **b** Cell invasive potential of transfected cells was measured with CytoSelect™ 24-Well Cell Invasion assay with type I collagen-based matrix. The invaded cells were stained with CyQuant^®^ GR dye and quantified in a fluorescence plate reader. The data were presented as mean ± SD of at least three independent experiments (*p < 0.05, **p < 0.01, ***p < 0.001 as compared with control)

Since miR-107 expression was tightly correlated with the progression of melanoma, we next analysed its effect on melanoma cell migratory and invasive potentials. To evaluate the impact on melanoma cell migration, we employed a wound healing model where an artificial wound was created in the centre of a confluent cell monolayer. We measured the wound closure rate over 3 days. As shown in Fig. 3a, control cells closed the open wound very efficiently. Around 50% wound closure was seen 24 h after the wound was created. In comparison, the transfected cells showed a much slower rate to recover the wound site. Only 50% recovery was seen 48 h after wounding. In day 3 nearly 100% of the wound site was covered by control cells. While there was still more than 20% open wound area in the cells transfected with miR-107. This result indicated miR-107 impacted on the migratory potential of melanoma cells. We also measured the impact of miR-107 on cell invasive potential. A type I collagen-based matrix was used to access the invasive property of transfected cells. As shown in Fig. 3b, cells expressing miR-107 exhibited about 40% reduction in their invasive activity into the collagen matrix as quantified by fluorescence staining.

Next, to examine if miR-107 carried anti-melanoma activity in vivo, we employed a xenograft model. We injected B16 melanoma cells to the flanks of BALB/c nude mice to induce tumor growth. When the tumors reached about 150 mm^3^, we treated the tumors with either scramble miRNA or miR-107 for about another 2 weeks. As shown in Additional file 1: Figure S2A, the tumor size from miR-107-treated site was considerably smaller. We also harvested the tumors for weigh measurement. As shown in Additional file 1: Figure S2B, the average tumor weight was markedly lower in miR-107 treated group. Therefore, in vivo xenograft experiment also supported that miR-107 inhibited melanoma growth.

**Fig. 4:**
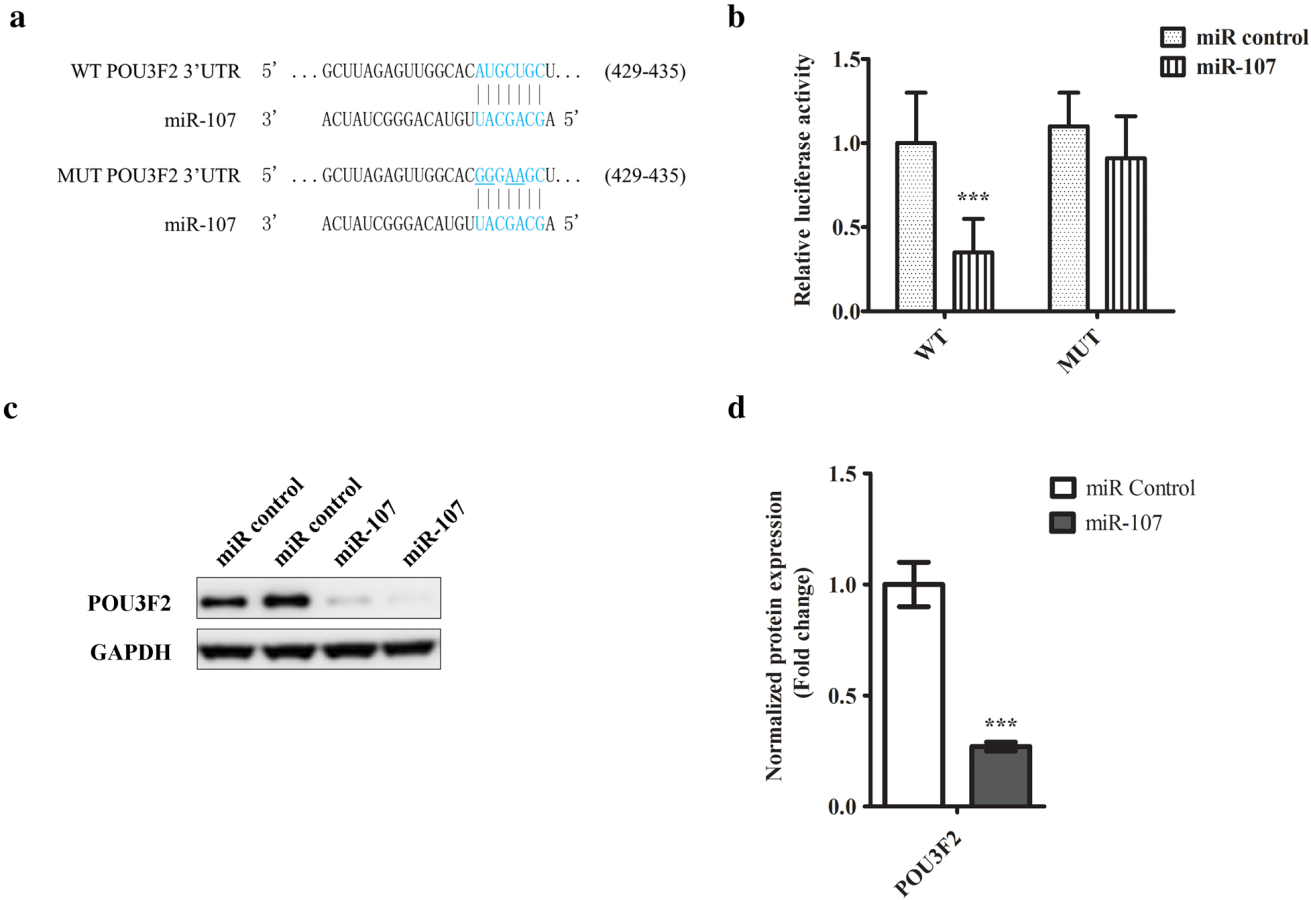
POU3F2 is a downstream target of miR-107 in melanoma cells. **a** Schematic diagram of a conserved putative miR-107 targeting site in 3′UTR of POU3F2 (WT). The site mutations were introduced to miR-107 targeting site of 3′UTR (MUT). **b** Luciferase reporter assay study of the interaction between miR-107 and 3′UTR of POU3F2. SH-4 cells were transfected with luciferase reporter genes conjugated with either wildtype (WT) or mutant (MUT) 3′UTR of POU3F2. 24 h later, the cells were transfected further with either miR-107 mimic or scramble control. Transfected cells were cultured for 48 h and then harvested for the measurement of luciferase activities in a plate reader. **c** Western blotting analysis of POU3F2 in cells transfected with either miR-107 or control miRNA. The representative image from at least three independent experiments was shown. **d** The densitometric analysis of POU3F2 expression was shown. The data were presented as mean ± SD of at least three independent experiments: (*p < 0.05, **p < 0.01, ***p < 0.001 as compared with control)

So far, we have established that miR-107 may act as a tumor suppressor in melanoma. Next, we proceeded to examine its target signalling pathway. Based on miRNA target prediction [15], a conserved binding site was found in the 3′UTR of POU3F2, a transcription factor found regulating invasiveness and proliferation of melanoma cells in a recent study [16]. The predicted binding site was shown in Fig. 4a. To test the binding, we cloned the sequence into the downstream of a luciferase gene in WT vector. To make the MUT vector, we mutated the conserved binding site on the cloned sequence. Subsequently, we transfected WT with miR-107 or miRNA control in SH-4 cells. As shown in Fig. 4b, the luciferase activity was largely suppressed by miR-107, suggesting the binding and inhibition of cloned luciferase gene transcript. However, miR-107 transfection failed to inhibit the luciferase in MUT vector, strongly suggesting the predicted binding site was required for the interaction. We also measured the inhibition of miR-107 on POU3F2 protein expression, and found that transfection of miR-107 but not scramble control downregulated the protein expression of POU3F2 in SH-4 cells (Fig. 4c, d).

**Fig. 5:**
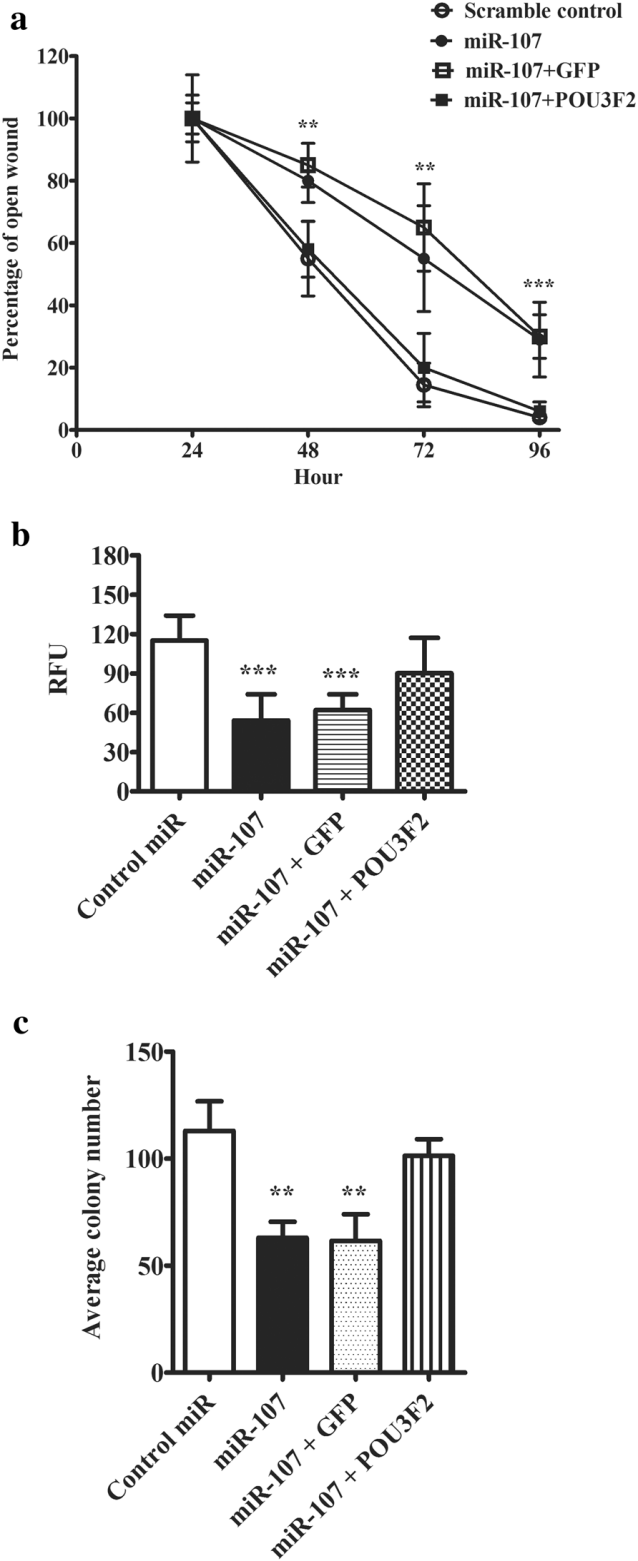
Over-expression of POU3F2 antagonized miR-107 inhibitory effects on melanoma cells. **a** Graphic summary of cell migration rate in wound closure experiment. SH-4 cells were co-transfected with miRNAs and POU3F2 or GFP. An open wound was created in the centre of a confluent monolayer of transfected cells. The percentage of the remaining open wound was recorded every 24 h for 4 days. **b** Cell invasive potential of transfected cells was measured with CytoSelect™ 24-Well Cell Invasion assay with type I collagen based matrix. SH-4 cells were co-transfected with miRNAs and POU3F2 or GFP. The transfected cells were plated on the upper chamber of a tran-well system. 48 h later, the matrix was collected and the invaded cells were stained with CyQuant^®^ GR dye and quantified in a fluorescence plate reader. **c** Colony formation study of SH-4 cells co-transfected with miRNAs and POU3F2 or GFP. The colony formation ability of the transfected cells was accessed by counting the number of colonies (more than 50 cells) in the culturing plates. The data were presented as mean ± SD of at least three independent experiments (*p < 0.05, **p < 0.01, ***p < 0.001 as compared with control)

Last but least, we addressed whether POU3F2 was required for miR-107-mediated suppression of melanoma cells. In the open wound closure assay, transfection of POU3F2 was shown to abolish the inhibitory effect of miR-107 on melanoma cell migration rate (Fig. 5a). But GFP co-transfection failed to do so. Similarly, co-expressing POU3F2 also rescued the reduction of cell invasiveness mediated by miR-107 (Fig. 5b). In cell colony formation test, POU3F2 was also shown to restore the colony formation potential in cells transfected with miR-107 (Fig. 5c). To produce the clinical insight of POU3F2 in melanoma, we moved on to compare its expression among three clinical sample groups: nevi from non-tumor tissues, primary melanoma and metastatic melanoma. As shown in Additional file 1: Figure S3, POU3F2 protein expression was significantly upregulated in both primary melanoma and metastatic tumors. There was even a higher expression difference in metastatic tumor than primary melanoma. Interestingly, the POU3F2 expression pattern in melanoma tissues was inversely correlated with miR-107. Taken together, these tests indicated POU3F2 was a functional downstream target of miR-107 in melanoma.

## Discussion

**Fig. 6:**
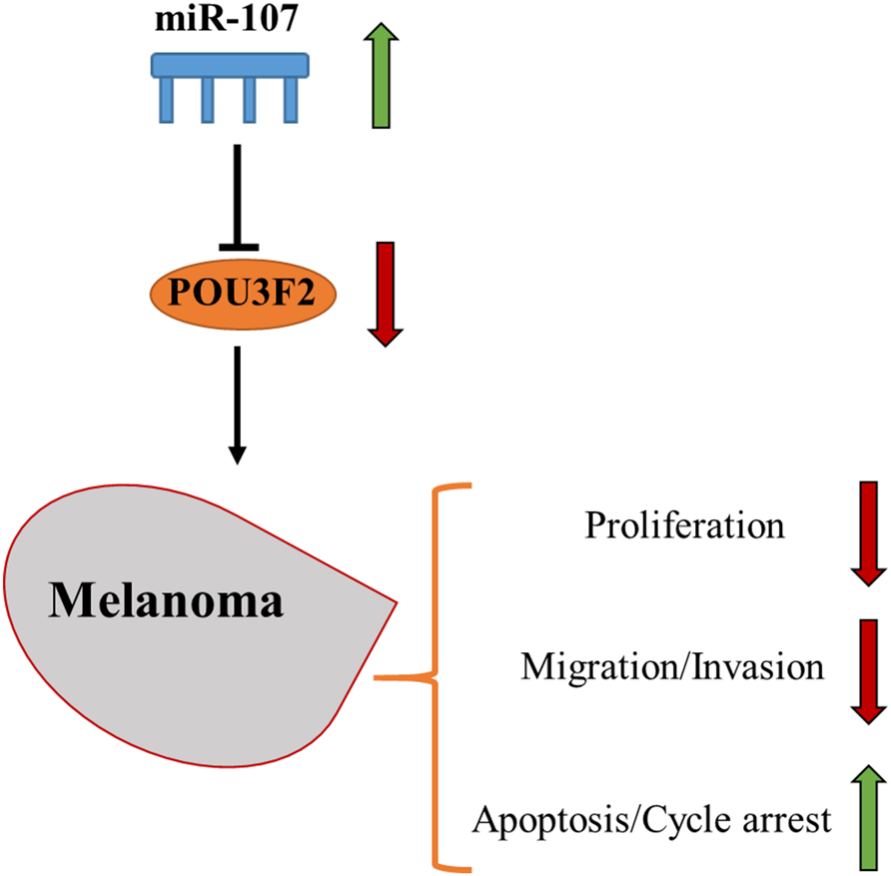
Schematic diagram of proposed mechanism. miR-107 expression may regulate melanoma cell proliferation, migration, invasion and apoptosis by inhibiting the expression of POU3F2

We are the first to uncover a significant role of miR-107 in melanoma. Its expressional level was suppressed in melanoma samples. The suppression degree was correlated with the severity of the tumor stages. The downregulation was most severe in metastatic melanoma. Functional characterization revealed that the exogenous expression of miR-107 in melanoma cells inhibited the tumor cell phenotypes including growth, migration and invasion. These results have strongly supported that miR-107 is a novel tumor suppressor in melanoma (Fig. 6).

The role of miR-107 in cancer has surfaced recently and the evidence is accumulating through the studies in different types of cancers. Its implication in tumorigenesis is firstly reported in the association with p53-dependent pathways. In human colon cancer model, the expression of miRNA is found to be regulated by p53 [17]. The induction of miR-107 suppresses hypoxia signalling by targeting hypoxia inducible factor-1β (HIF-1β). Downregulation of HIF-1β leads to the reduced production of VEGF that eventually contributes to decreased angiogenesis in mouse tumor model. This axis is proposed as the downstream mechanism of p53-induced tumor angiogenesis suppression. In another study, miR-107 was proposed to mediate p53-dependent cell cycle regulation [18]. By microarray screening the authors identified a list of p53-regulated genes containing intronic miRNAs. Among the list miR-107 was reported as an intronic miRNA of panthothenate kinase 1 (PANK1). The study has used different cell systems to establish that the induction of PANK1 and miR-107 is regulated by p53. And subsequent functional characterization has revealed that miR-107 regulates the expression of RB-related 2 gene RBL2 (p130) and CDK6, two important players in G1/S transition. Therefore, the induction of miR-107 by p53 can also contribute to the cell cycle regulation, a key step in malignant cancer formation. Frequent mutations in p53 have been associated with skin cancers caused by UV exposure. A subset of melanoma also harbours mutations on the p53 gene [19]. It is hypothesized that the suppression of miR-107 could be p53 pathway dependent. It would be intriguing to explore if the low expression of miR-107 is correlated with the level or mutation rates of p53.

In addition, miR-107 was found decreased in breast cancer specimens. In breast cancer, the miRNA targets CDK8 in the cell cycle pathway [20]. In renal clear cell carcinoma, miR-107 was also confirmed downregulated in clinical specimens [21]. Over-expressing miR-107 induces the arrest of renal carcinoma cells in G2/M phase by targeting multiple genes. In non-small cell lung cancer, miR-107 was proposed as a drug target to enhance the chemosensitivity of paclitaxel [22]. In this model, miR-107 was found directly inhibiting anti-apoptotic factor Bcl-w. The treatment of miR-107 reverses drug resistance in both in vitro cell experiment and in vivo xenograft model. Therefore, our study in melanoma is consistent with these previous studies from different cancer models to support the role of miR-107 as a tumor suppressor. The functional characterization of our current study is largely done on cell model. It will be important for the next step to validate its role in melanoma in vivo using xenograft or UV induced mouse models. The mainstream of chemotherapy for melanoma is BRAF inhibitors [6]. It has shown promising outcome in advanced stage melanoma. However, the success is limited by the occurrence of drug resistance or the relapse of tumor growth. Alternative drug targets such as miR-107 may provide a promising approach to improve the chemosensitivity of melanoma to BRAF inhibitors. The similar concept was proposed in non-small cell lung cancer for overcoming paclitaxel resistance.

In our study, we have described the mechanism of miR-107 in inhibiting melanoma. POU3F2 has been identified as an endogenous target of miR-107. Over-expressing POU3F2 reverses the inhibitory effect of miR-107 on melanoma cells. And its protein expression in melanoma tissues is inversely correlated with miR-107 level. POU3F2, also known as BRN2, belongs to POU domain family of transcription factors. They play a critical part in embryogenesis. They are highly expressed in the brain and regulate the function and development of the central nervous system. The prominent members include Oct-1, Oct-2 and Pit-1 [23]. POU3F2 is a class III POU domain protein and firstly identified as a brain specific gene expressed during early embryogenesis [24]. It plays an important part in the development or differentiation of neural cells. For instance, POU3F2 acts with other key transcription factors, such as Oct6 and Sox10, to control myelination in the Schwann cells. Interestingly, melanocytes are originated from the neural crest and derived from the same ancestor cell population as the Schwann cells. Mounting studies have revealed a critical role of POU3F2 in melanocytic lineage development, melanocyte growth/activation and malignant transformation. A mechanistic study has reported phosphorylated POU3F2 can control melanocyte proliferation and migration through modulating its interaction with Pax3 and MITF-M, both of which are the critical regulators for the establishment of transformation of melanocyte lineage [25]. In melanoma, POU3F2 has been proposed as a key driver for tumour progression and metastasis [26]. Goodall et al. have firstly shown POU3F2 directly suppresses MITF expression and marks a distinct population of melanoma cells. A subsequent study has proposed both MITF and POU3F2 are critical for metastatic growth of melanoma in vivo. Their interplay contributes to the heterogeneity of melanoma and is strongly implicated in the cancer progression and tumor phenotype switching. For instance, POU3F2 represses MITF to drive cells to a more invasive phenotype. PI3K signalling is reported to act upstream of POU3F2 in melanoma. Inhibiting PI3K is shown to reduce tumor cell invasion through downregulating POU3F2 [27]. Interestingly, miR-211 was a tumor suppressive miRNA previously identified in melanoma [28]. Its functional downstream target was identified as POU3F2 in the same study. Inhibiting miR-211 leads to the increased POU3F2 and subsequently promotes invasive potential of melanoma cells.

## Conclusion

Together with the past studies, our work confirms the important function of POU3F2 in driving melanoma tumorigenesis and its metastatic transformation (Fig. 6). The newly identified miR-107-POU3F2 axis offers new therapeutic targets to manage metastatic melanoma.

## Supplementary information

**Additional file 1:** Supplementary Figures S1, S2, and S3.

## Data Availability

The data could be obtained upon request to the corresponding author.

## Abbreviations

MITF: Micropthalmia-associated transcription factor
miR: MicroRNA
FBS: Fetal bovine serum
CCK-8: Cell Counting Kit-8
HIF-1β: Hypoxia inducible factor-1β
PANK1: Panthothenate kinase 1
