# Supplementary material for: MicroRNA-107 is a novel tumor suppressor targeting POU3F2 in melanoma
Source: Biol Res. 2020 Mar 14;53:11. doi: 10.1186/s40659-020-00278-3 (PMC7071777; doi:10.1186/s40659-020-00278-3)
Supplement: Supplementary file 1 — Additional file 1: Supplementary Figures S1, S2, and S3. [file 40659_2020_278_MOESM1_ESM.docx]

**Additional Materials**

**Additional methods**

**Melanoma xenograft experiment**

Female BALB/c nude mice were purchased from Vital River Lab in China. A total of 6×10^6^ B16 cells in 200 µl PBS was subcutaneously injected into the flanks of 6-week old mice. When solid tumors were reached to about 150 mm^3^, *in vivo* delivery of jetPEI/miRNA complex (miR control or miR-107) was given randomly to either flank of the subject every three days. The delivery mixture contained 20 µg miRNA in 1.5 µl *in vivo*-jetPEI reagent in 100 µl 5% glucose. The mice were sacrificed in day 21 after cell implantation and the tumors excised and weighted.

**Additional data**


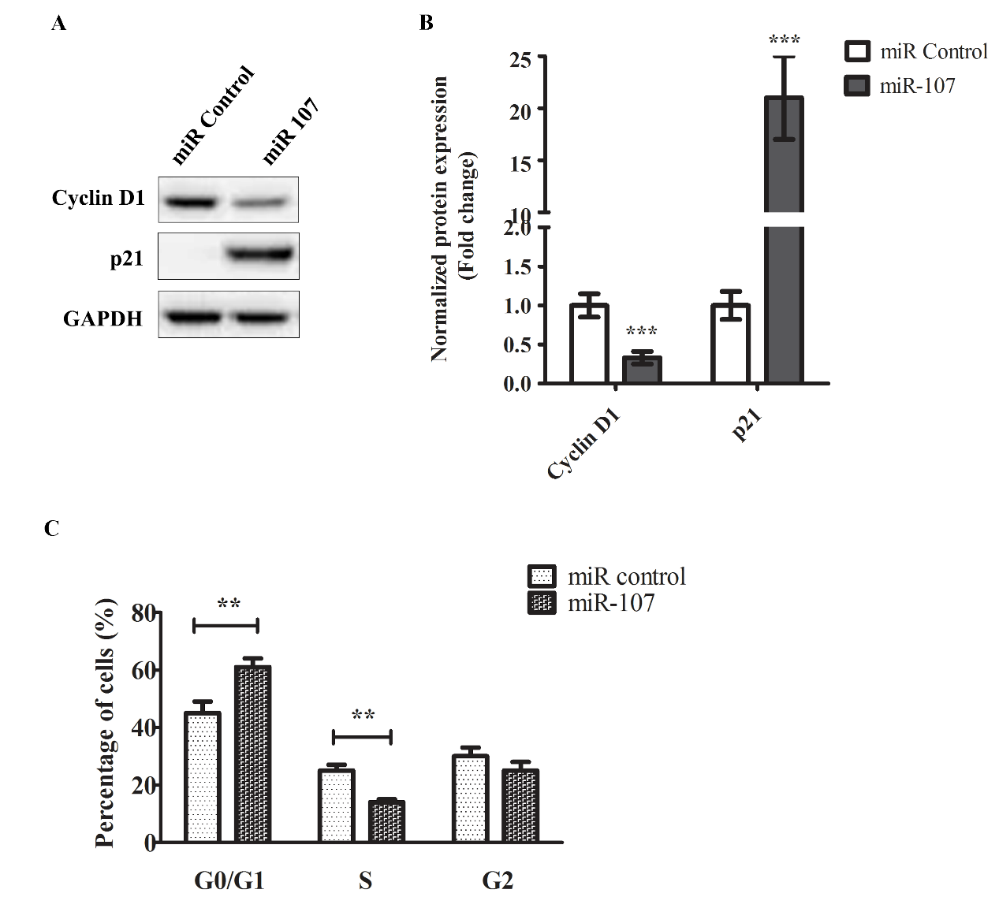


**Figure S1.** miR-107 induced cell cycle arrest in melanoma cells.

**A)** Western blot analysis of Cyclin D1 and p21 in SH-4 cells transfected with either miR-107 or the scramble control. The representative image from at least three independent experiments was shown. **B)** The densitometric analysis of Cyclin D1 and p21 expression was shown. **C)** Representative histogram data of the cell-cycle analysis of SH-4 cells transfected with either miR-107 or the scramble control. The data were presented as means ± S.D. (**p <0.01, ***p<0.001 as compared with control).


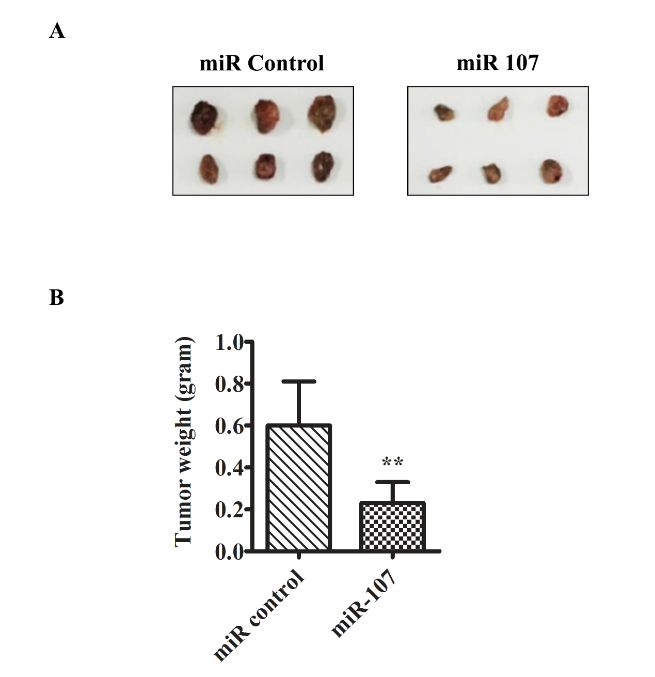


**Figure S2.** Antitumor activity of miR-107 in melanoma tumor model.

Mice (n=6) were sacrificed and tumors were harvested on 18^th^ day for **A)** image presentation and **B)** weight measurement. The data were presented as means ± S.D. (**p <0.01, as compared with control).


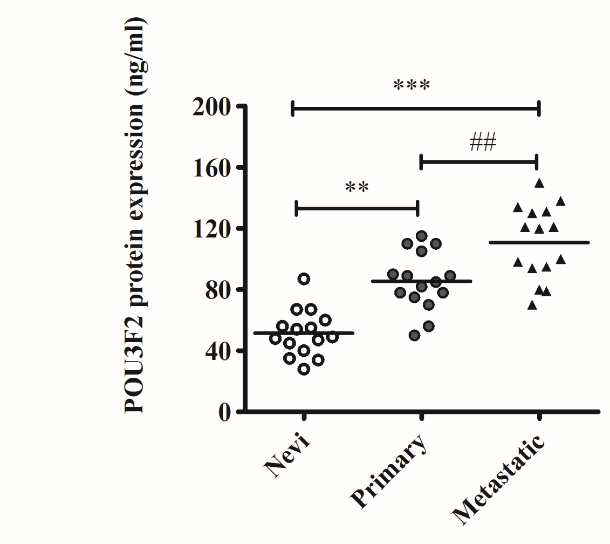


**Figure S3.** POU3F2 is over-expressed in human melanoma cell lines and tumors.

The comparison of POU3F2 expression in nevi, primary and metastatic melanoma human samples. The protein expression was quantified by ELISA. (** p<0.01, *** p<0.001, as compared with control cells or nevi tissues; ## p<0.05, as compared with primary tumors).
